# Diagnostic Performance of GPT-4o Compared to Radiology Residents in Emergency Abdominal Tomography Cases

**DOI:** 10.3390/tomography11100108

**Published:** 2025-09-26

**Authors:** Ahmet Tanyeri, Rıdvan Akbulut, Cuma Gündoğdu, Tuğba Öztürk, Büşra Ceylan, Nasır Fırat Yalçın, Ömer Dural, Selin Kasap, Mehmet Burak Çildağ, Alparslan Ünsal, Yelda Özsunar

**Affiliations:** Department of Radiology, Faculty of Medicine, Aydın Adnan Menderes University, Aydın 09010, Türkiye

**Keywords:** artificial intelligence, emergencies, abdomen, tomography, radiology

## Abstract

**Purpose:** This study aimed to evaluate the diagnostic performance of GPT-4 Omni (GPT-4o) in emergency abdominal computed tomography (CT) cases compared to radiology residents with varying levels of experience, under conditions that closely mimic real clinical scenarios. **Material and Methods:** A total of 45 emergency cases were categorized into three levels of difficulty (easy, moderate, and difficult) and evaluated by six radiology residents with varying levels of experience (limited: R1–R2; intermediate: R3–R4; advanced: R5–R6) and GPT-4o. Cases were presented sequentially to both groups with consistent clinical data and CT images. Each case included 4 to 7 CT slice images, resulting in a total of 243 images. The participants were asked to provide the single most likely diagnosis for each case. GPT-4o’s CT image interpretation performance without clinical data and hallucination rate were evaluated. **Results:** Overall diagnostic accuracy rates were 76% for R1–R2, 89% for R3, 82% for R4–R5, 84% for R6, and 82% for GPT-4o. Case difficulty significantly affected the diagnostic accuracy for both the residents and GPT-4o, with accuracy decreasing as case complexity increased (*p* < 0.001). No statistically significant differences in diagnostic accuracy were found between GPT-4o and the residents, regardless of the experience level or case difficulty (*p* > 0.05). GPT-4o demonstrated a hallucination rate of 75%. **Conclusions:** GPT-4o demonstrated a diagnostic accuracy comparable to that of radiology residents in emergency abdominal CT cases. However, its dependence on structured prompts and high hallucination rate indicates the need for further optimization before clinical integration.

## 1. Introduction

The integration of artificial intelligence (AI) into healthcare has advanced at a remarkable pace, with significant milestones, such as the adoption of machine learning algorithms for diagnostic imaging and the development of predictive models for patient care. Among these innovations, generative pre-trained transformers (GPTs) have emerged as powerful tools for reshaping medical practices. GPT-4o, the latest iteration of this technology, was released in May 2024 and has promising new capabilities in various applications (https://chatgpt.com/, accessed on 5 January 2025).

Radiology has rapidly adopted advancements in image processing, algorithm development, and data analytics because of its strong reliance on technology [1]. Numerous studies have explored the potential of the GPT-4o, demonstrating its utility in improving clinical documentation, enhancing educational resources, generating structured radiology reports, drafting personalised recommendation letters, simplifying patient education, and assisting in disease screening, diagnosis, and monitoring [2,3,4,5,6,7,8]. These studies underscore the possibility of GPT-4o streamlining radiological workflows and enhancing overall efficiency. However, while some argue that GPT-4o can alleviate the radiologist workload and improve diagnostic accuracy [9], others caution that the technology is still evolving and may occasionally lead to significant errors in interpretation [10].

As computational capabilities continue to grow and the demand for efficient healthcare solutions increases, the role of GPT-4o and similar AI systems in radiology is expected to expand. For this expansion to be meaningful, ensuring that these technologies are integrated into clinical practice in a manner that is reliable, ethical, and collaborative, is crucial. Testing AI systems, such as GPT-4o, in realistic clinical scenarios is a key step toward this goal. Previous studies have evaluated the diagnostic performance of GPT-based models in various radiology subspecialties, often in retrospective or report-based formats rather than real-world clinical scenarios. Many lacked direct comparison with human readers of varying expertise levels, especially in emergency abdominal imaging. To the best of our knowledge, this study is among the earliest in the literature to address this gap. The aim of this study was to evaluate the performance of GPT-4o in interpreting emergency abdominal radiological examinations and to compare its diagnostic accuracy with that of radiology residents at different levels of expertise under conditions designed to closely mimic real clinical practice.

## 2. Material and Methods

### 2.1. Study Design

This study was approved by the local ethics committee (date: 24 October 2024, no. 632228). The requirement for individual informed consent was waived because of the use of retrospective and anonymized data. The study was designed as a prospective evaluation of retrospectively selected abdominal cases, with assessments conducted by radiology residents and GPT-4o. Six radiology residents (R1–R6) with varying levels of experience and GPT-4o were tasked with interpreting 45 emergency radiological cases. The cases were classified into three categories based on difficulty: easy, moderate, and difficult, with 15 questions in each group. The sequence of questions was randomized to eliminate potential bias.

For residents, each case was presented on an individual slide deck. The consistent format of the slide decks was designed to standardize the presentation of cases, ensure fairness, and minimize variability in information delivery. The first slide contained the patient’s clinical and laboratory data, followed by the radiological images in subsequent slides. The final slide posed the following question: *“Based on the patient’s data and radiological images, what is the single most likely diagnosis?”* Each participant individually evaluated the cases under the guidance of a supervisor who ensured adherence to the study protocol and consistent data collection. No time limitations were imposed during the evaluations, and the supervisor did not proceed to the next case until all residents confirmed that they had completed their assessments. Once a case is complete, it cannot be revisited.

**Figure 1:**
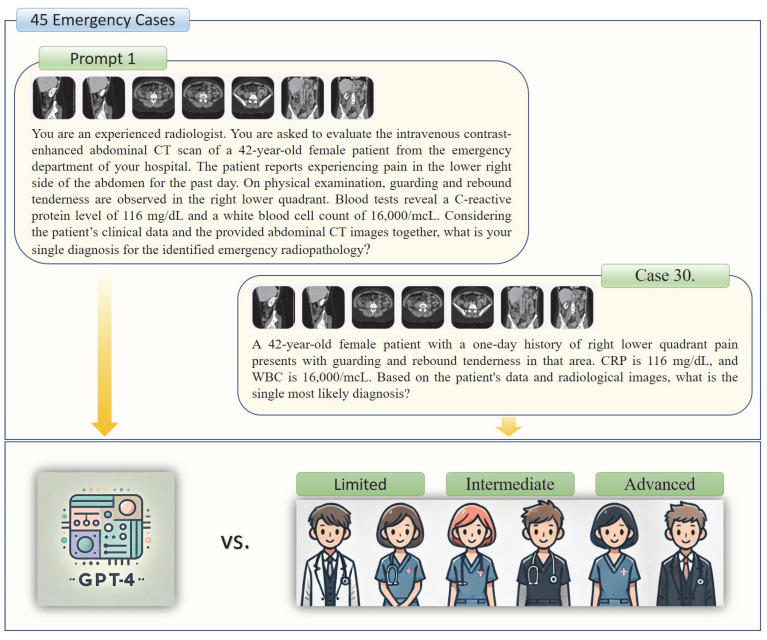
The study group participants and a presented emergency case example are illustrated.

GPT-4o was evaluated approximately one week prior to the residents using the same sequence of cases. Each case was presented in a separate session [11], with radiological images and prompts provided within the same dialogue interface. The flow diagram of the study is shown in Figure 1.

### 2.2. Radiology Residents

In our country, radiology residency training spans five years and follows a structured rotation program accredited by the National Radiology Accreditation Board at our institution. First-year residents participated in an intensive emergency radiology training program that covered all imaging modalities. After completing their first year, the residents started participating in on-call duties at the hospital.

During on-call shifts, two radiology residents—a junior (with less experience) and a senior (with more experience)—collaborate to manage emergency cases. The junior resident conducted the initial assessment of the radiological images and drafted a preliminary report. The senior resident reviewed the findings, revised the report if needed, and consulted a supervising radiologist when necessary. If no further consultation is needed, the senior resident finalizes and approves the report.

This study involved six radiology residents who had completed their first year of training and had diverse levels of experience. Residents were classified into three groups based on their experience: limited experience (1–2.5 years), moderate experience (2.5–4 years), and advanced experience (4–5 years).

### 2.3. Case Selection and Preparation

The cases were selected and prepared collaboratively by two board-certified radiologists with 12 and 25 years of experience. A retrospective review was conducted of abdominal CT scans obtained from the emergency department of our institution between 2017 and 2019. This period was chosen to ensure the inclusion of cases that predated the tenure of our senior radiology residents. All examinations were performed using a single imaging device (Prime Aquilion, Toshiba Medical Systems, Otawara, Japan).

A total of 45 cases were selected and categorized into three groups based on difficulty level: easy, moderate, and difficult, with 15 cases in each group. This classification is based on the synthesis of clinical and radiological features, such as the frequency of occurrence, patient history, physical examination findings, and laboratory results. Cases were excluded if they had non-diagnostic quality CT images, multiple overlapping emergency findings, or incomplete/erroneous clinical data. Relevant case data were retrieved from the hospital information and management system. The cases are listed in Table 1.

For each patient, the most representative CT slices highlighting the emergency radiological findings were anonymized after adjusting the window settings based on the anatomical region and nature of the findings. The images were saved in the JPEG format, and no modifications, such as zooming, cropping, or labelling, were made.

### 2.4. GPT-4o Prompts

**Figure 2:**
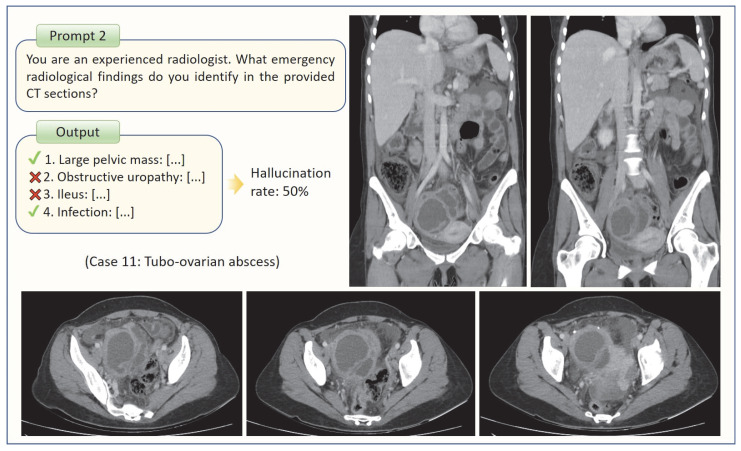
The method used to determine GPT-4o’s hallucination rate is illustrated through an example case.

Two distinct prompts (‘Prompt 1’ and ‘Prompt 2’) were provided to GPT-4o for each patient. Prompt 1 assessed GPT-4o’s diagnostic performance by combining clinical and radiological findings, whereas Prompt 2 evaluated its ability to interpret radiological findings and identify potential hallucinations. Both prompts started with the following statement: *“You are an experienced radiologist. You are asked to evaluate the abdominal CT scan of a [X]-year-old male/female patient from the emergency department of your hospital.”* Prompt 1 incorporated clinical information including the patient’s presenting complaint, physical examination findings, and laboratory test results. The concluding question of Prompt 1 asked: *“Considering the patient’s clinical data and the provided abdominal CT sections together, what is your single diagnosis for the identified emergency radiopathology?”* Conversely, Prompt 2 included the following question: *“You are an experienced radiologist. What emergency radiological findings do you identify in the provided CT sections?”* The same CT sections, presented in the same sequence, were used for both Prompt 1 and Prompt 2 for each case. The two trials were conducted at different times in separate sessions to avoid potential carryover effects. The outputs were recorded individually for each case. Figure 1 illustrates an example case corresponding to Prompt 1, whereas Figure 2 provides a representative example for Prompt 2.

### 2.5. Evaluation of Answers and Outputs

The answers and outputs were collaboratively reviewed by two experienced radiologists who classified them as either correct or incorrect. Although each question had a specific correct answer, responses that captured the essence of the correct answer, conveyed the same meaning, or provided an accurate yet differently phrased explanation were considered correct. For example, in cases where the correct answer was gastrointestinal perforation, the response to pneumoperitoneum was also considered correct. Similarly, both “acute cholecystitis” and “acute calculous cholecystitis” were deemed correct. Unanswered questions by radiology residents were incorrectly classified.

For Prompt 1, GPT-4o provided a single response per question, whereas for Prompt 2, it listed the emergency radiopathological findings it identified. The radiologists re-evaluated the images to validate the accuracy of these findings and subsequently calculated the false positive rate. For example, if GPT-4o listed four findings, of which two were correct and two were incorrect, the false-positive rate (hallucination rate) was calculated to be 50% (Figure 2). In this study, hallucinations were strictly defined as findings reported by GPT-4o that had no radiological correlate on the uploaded CT slices. However, if GPT-4o described a finding that could not be definitively confirmed or excluded based on the available slices, such outputs were regarded as neutral and were not included in the calculation.

### 2.6. Statistical Analysis

All statistical analyses were performed using SPSS version 26 (IBM Corp., Armonk, NY, USA). Descriptive statistics, including accuracy rates, were calculated for each participant and grouped according to experience level. Diagnostic performance comparisons were performed using chi-square tests to evaluate the differences in accuracy between GPT-4o and radiology residents across various experience levels.

To assess the impact of case difficulty on the diagnostic accuracy, a logistic regression model was applied. Case difficulty was included in the model as an ordinal categorical variable (easy = 1, moderate = 2, difficult = 3), with the easy group set as the reference category. Dummy variables were generated accordingly. Diagnostic accuracy (1 = correct, 0 = incorrect) was used as the binary dependent variable, and participant group was entered as a categorical covariate with GPT-4o as the reference. For all analyses, a *p* value of <0.05 was considered to indicate statistical significance.

## 3. Results

### 3.1. Diagnostic Performance

**Figure 3:**
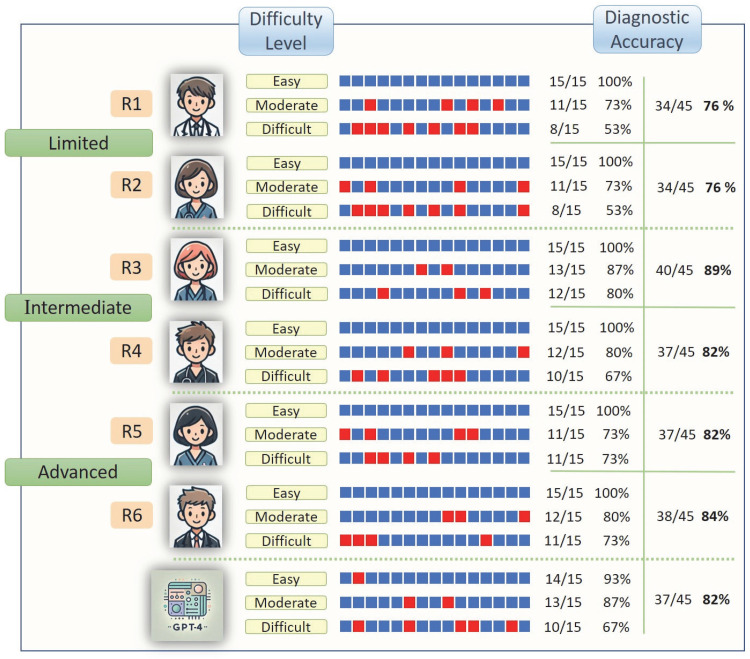
The diagnostic accuracy rates of the study group participants, on the basis of their responses categorised by question difficulty level, are illustrated. The blue boxes represent correct answers, whereas the red boxes represent incorrect answers.

The dataset included 45 cases, each consisting of 4–7 CT slice images, for a total of 243 images. R3 demonstrated the highest diagnostic performance among the six residents, with an accuracy of 88.89%. The remaining residents had the following accuracy rates: R6, 84.44%; R4 and R5, 82.22%; and R1 and R2, 75.56%. GPT-4o achieved an accuracy of 82.22%, ranking second overall, and performing comparably to R4 and R5. When the data were grouped by experience level, the limited-experience groups (R1 and R2) presented the lowest accuracy at 75.56%. The intermediate-experience group (R3 and R4) achieved an accuracy of 85.56%, whereas the advanced-experience groups (R5 and R6) attained 83.33% accuracy. Figure 3 provides a summary of the diagnostic accuracy rates categorized by the case difficulty level. The total hallucination rate of GPT-4o was calculated to be 75%. When hallucination rates were analyzed according to case difficulty, GPT-4o demonstrated a rate of 72% in easy cases, 75% in moderate cases, and 78% in difficult cases. Although the rates tended to increase with case complexity, the differences between groups were not statistically significant.

### 3.2. Comparative Diagnostic Accuracy

The diagnostic accuracy rates in 45 emergency radiology cases were compared between GPT-4o and six radiology residents. Chi-square tests indicated no significant differences in diagnostic accuracy between GPT-4o and radiology residents across experience levels: limited (*p* = 0.605), intermediate (*p* = 0.549), or advanced (*p* = 1.000).

### 3.3. Impact of Case Difficulty on Diagnostic Accuracy

**Figure 4:**
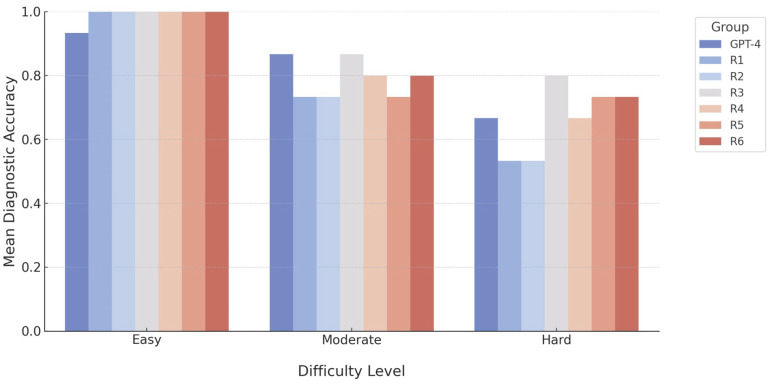
A bar chart demonstrating the diagnostic accuracy of GPT-4o and radiology residents across varying levels of question difficulty.

Logistic regression analysis indicated a significant impact of case difficulty on the diagnostic accuracy (χ^2^ = 26.39, *p* < 0.001, pseudo-R^2^ = 0.1755). Compared with easy cases, moderate-difficulty cases were associated with a reduction in correct diagnoses (β = -3.34, 95% CI: −5.37 to −1.31, *p* = 0.001), whereas difficult cases further decreased accuracy (β = -3.99, 95% CI: −6.00 to −1.97, *p* < 0.001). No significant differences were detected among the participant groups, including the radiology residents (R1–R6) and GPT-4o groups (*p* > 0.05). Although R3 demonstrated a slightly positive trend compared with GPT-4o (β = 0.60, 95% CI: −0.66 to 1.86, *p* = 0.349), this difference was not statistically significant (Figure 4).

## 4. Discussion

This study is among the first to evaluate and compare the diagnostic performance of GPT-4o in emergency radiology cases with that of radiology residents with varying levels of experience. These findings suggest that the diagnostic accuracy of GPT-4o is comparable to that of radiology residents, particularly in intermediate and advanced experience groups. However, the high hallucination rate observed in this study poses critical challenges to the applicability, reliability, and safety of AI systems in clinical practice.

Artificial intelligence relies on mathematical and statistical methods to learn from data and make predictions or decisions in novel scenarios. Rigorous testing, simulation, and deployment in real-world scenarios are essential to assess their capabilities and limitations. In a recent review, Keshavarz et al. reported that as of 1 January 2024, 44 studies on the application of GPT in clinical radiology have been published, 20 of which focused specifically on diagnostic and clinical decision support systems. However, many of these studies are distant from practical clinical applications and lack real-world validations.

The use of structured and detailed prompts has been shown to increase the diagnostic accuracy of AI systems [1,12,13,14]. In this study, we aimed to optimize AI diagnostic performance by using a single, structured, and standardized model for each case in Prompt 1. The results revealed an intriguing finding: GPT-4o demonstrated behavior and performance patterns similar to those of human reasoning. For human assistants, the diagnostic performance is expected to decrease as the case complexity increases. The observation of the same trend for GPT-4o suggests that it can emulate human-like reasoning abilities. Several recent studies have compared GPT-4’s diagnostic performance of GPT-4 with that of human experts. For example, Mitsuyama et al. reported that the GPT-4 achieved a diagnostic accuracy of 94% in identifying findings from 150 brain tumour cases, surpassing that of general radiologists and neuroradiologists [15]. On the other hand, Horiuchi et al. reported that the performance of the GPT-4 in diagnosing 32 clinical neuroradiology cases was lower than that of three radiology residents [11]. Siepmann et al. conducted a study involving 40 radiology cases and six residents with varying levels of experience. They concluded that integrating GPT-4 into the diagnostic process slightly improved accuracy but significantly increased diagnostic confidence [13]. This study differs from previous studies by employing a novel methodological approach to test AI performance in clinical scenarios that closely mimic real-world conditions. Interpreting emergency radiological examinations is often a challenging task for radiology residents because the need for speed combined with limited experience can increase error rates. At our institution, first-year residents underwent intensive training in emergency radiology, resulting in acceptable diagnostic accuracy rates of 76% among the less experienced group in our study. However, similar to many other countries, standardization of radiology training in our country remains incomplete and inconsistent. In several institutions, less-experienced residents are often required to manage on-call duties independently. In this context, GPT-4’s diagnostic accuracy of 82% in emergency abdominal CT examinations, achieved through the use of well-structured prompts, demonstrates its potential as a valuable tool to support clinical decision-making in real-world applications.

GPT-4o shows promise for analyzing clinical data for diagnostic purposes; however, its inability to independently interpret radiological images is a significant limitation. This phenomenon, called hallucination, occurs when AI generates incorrect, fabricated, or contextually irrelevant information. Recent studies have explored this issue and reported high hallucination rates across imaging modalities. Brin et al. reported the highest rate of ultrasonography (60.6%), followed by CT (51.5%) and X-ray imaging (19.6%) [16]. Similarly, Huppertz et al. reported rates of 96.7% for both CT and MRI, 88.5% for angiography, and 83.3% for X-ray [13]. While these variations highlight the influence of modality and methodology, the consensus is that GPT-4 cannot reliably perform image-based diagnostics. Our findings, which revealed a 75% hallucination rate, align with the broader literature. Unlike radiology residents, who may sometimes reach accurate conclusions using incomplete or erroneous clinical data, GPT-4o’s reliance solely on image inputs makes it incapable of achieving a comparable diagnostic accuracy. This underscores the urgent need for advancements to bridge the gap between the potential of AI and practical clinical utility.

In addition to these methodological considerations, the ethical aspects of applying large language models in clinical radiology must also be addressed. Patient safety remains the foremost priority, and AI-generated outputs should always be reviewed under expert supervision to prevent potential harm from diagnostic errors. The question of liability for AI-related mistakes also remains unresolved within current legal and regulatory frameworks. Moreover, while such tools may provide valuable support in resident training and clinical workflows, they should complement rather than replace human judgment, and their implementation requires structured oversight to avoid overreliance.

This study had several limitations. Although the selected radiology residents and cases did not comprehensively represent the broader clinical population, the sample group was carefully designed to closely simulate real-world clinical scenarios. However, the ideal nature of the clinical data, image quality, and case presentation differs from those of routine practice, potentially introducing a source of bias into the findings. Although the prompts used in this study were designed based on previous literature to best demonstrate the diagnostic performance of GPT-4o, minor changes in prompt structure may influence diagnostic accuracy. As this was not the primary focus of the study, this factor was not tested. While this approach ensured standardization, it does not evaluate potential performance variations with alternative prompt designs. Future studies should investigate a broader range of prompt structures to assess their influence on diagnostic accuracy.

## 5. Conclusions

This study evaluated the diagnostic performance of GPT-4o in emergency abdominal radiology cases by comparing it with that of radiology residents with varying experience levels, and demonstrated that the model achieved accuracy rates comparable to those of residents. However, its high hallucination rate and reliance on structured prompts highlights its current limitations as an independent diagnostic tool. Nonetheless, GPT-4o’s ability to exhibit human-like decision-making behaviour suggests that when supported with accurate and well-structured prompts, it could serve as a valuable aid in clinical decision support systems. We conclude that GPT-4o, in its current form, should be utilized not as a standalone diagnostic tool, but as a support system overseen by experienced radiologists.

## Figures and Tables

**Table 1:** Difficulty levels, diagnoses, and clinical descriptions of emergency radiology cases.

Case No.	Difficulty Level	Diagnosis	Summary Clinical Description
1	Moderate	Pneumatosis intestinalis	A 91-year-old male with sudden severe abdominal pain, rebound, leukocytosis, elevated lactate, and metabolic acidosis.
2	Difficult	Intussusception	A 51-year-old male with 12 h intermittent abdominal cramping, nausea, vomiting, periumbilical tenderness, and normal lab results.
3	Difficult	Omental infarction	A 31-year-old female with 24 h right lower quadrant pain, worsened by movement, local tenderness, mild guarding, no rebound, normal leukocyte count, and slightly elevated CRP.
4	Difficult	SMA thrombosis	An 88-year-old woman with sudden severe abdominal pain, elevated lactate, D-dimer, metabolic acidosis, and atrial fibrillation.
5	Difficult	Contained perforation	A 77-year-old female with 10-day abdominal pain, sudden relief followed by localized left lower abdominal pain, rebound tenderness, guarding, leukocytosis, and metabolic acidosis.
6	Difficult	Aortic aneurysm rupture	A 73-year-old male with sudden severe abdominal and back pain, hypotension, tachycardia, tachypnea, tenderness, and guarding. Blood tests show low hemoglobin.
7	Difficult	Postoperative bilioma	A 28-year-old female with right upper quadrant pain, jaundice, hepatomegaly, elevated bilirubin, GGT, alkaline phosphatase, and low albumin post-gallbladder surgery.
8	Difficult	Ovarian cyst rupture	A 23-year-old female with sudden pelvic pain during intercourse, tenderness, guarding, and low hematocrit.
9	Moderate	Liver laceration	A 39-year-old male with right upper abdominal pain, tenderness, redness, hypotension, tachycardia, low hemoglobin, and mildly elevated liver enzymes after a fall from height.
10	Easy	Psoas abscess	A 62-year-old male with low back and groin pain, fever, chills, lumbar tenderness, mild groin swelling, leukocytosis, elevated CRP, and mild anemia.
11	Moderate	Tubo-ovarian abscess	A 31-year-old female with fever, groin pain, foul-smelling vaginal discharge, pelvic tenderness, elevated leukocytes, CRP, and sedimentation.
12	Easy	Pneumoperitoneum	A 30-year-old male with sudden severe epigastric pain, rebound, hypotension, leukocytosis, and metabolic acidosis.
13	Moderate	Sigmoid volvulus	A 76-year-old male with abdominal distension, severe pain, stool and gas stasis, chronic constipation, leukocytosis, and mild lactate elevation.
14	Moderate	Renal artery occlusion	A 72-year-old male with sudden right flank pain, atrial fibrillation, costovertebral tenderness, and elevated lactate, D-dimer, and creatinine.
15	Difficult	Diverticulitis perforation	A 51-year-old male with diffuse abdominal pain, high fever, left lower quadrant rebound, hypotension, tachycardia, leukocytosis, elevated CRP, and lactate.
16	Difficult	Ovarian torsion	A 26-year-old female with sudden severe left lower quadrant pain, tenderness, guarding, and rebound, with normal blood tests.
17	Easy	Ileus	A 41-year-old male with abdominal pain, severe nausea, vomiting, 5-day absence of gas and stool, and abdominal tenderness.
18	Easy	Pyelonephritis	A 20-year-old female with right-sided pain, fever, costovertebral tenderness, and positive urine findings.
19	Difficult	Paraduodenal hernia	A 64-year-old female with abdominal pain, bloating, left upper quadrant swelling, and elevated lactate.
20	Easy	Umbilical hernia	A 44-year-old male with painful umbilical swelling, redness, and tenderness after heavy lifting, with normal blood tests.
21	Moderate	Liver abscess	A 69-year-old woman with 2-day right upper abdominal pain, high fever, nausea, loss of appetite, tenderness in the right upper quadrant, elevated leukocytes, and CRP.
22	Moderate	Portal vein thrombosis	A 42-year-old male with epigastric and right upper quadrant pain, nausea, leukocytosis, elevated D-dimer, mild liver abnormalities, and recent abdominal surgery.
23	Difficult	Epiploic appendagitis	A 61-year-old female with sharp left lower abdominal pain, tenderness, rebound, normal temperature, mildly elevated CRP, and borderline high white blood cell count.
24	Moderate	Pancreatic transection	A 24-year-old male with abdominal trauma after a motorcycle accident, epigastric tenderness, guarding, ecchymoses, elevated amylase and lipase, mild leukocytosis, and low hematocrit.
25	Moderate	SMV thrombosis	A 55-year-old woman with abdominal pain, tenderness, elevated D-dimer, mildly increased lactate, recent surgery, and stopped anticoagulants.
26	Moderate	Gastric perforation	A 22-year-old male with an epigastric stab wound, pain, guarding, rebound, tachycardia, and low hemoglobin.
27	Easy	Acute pancreatitis	A 63-year-old male with epigastric pain, nausea, and elevated amylase, lipase, and CRP.
28	Easy	Inguinal hernia	A 62-year-old male with left groin pain and reducible inguinal swelling, worsened by standing or coughing.
29	Easy	Diverticulitis	A 60-year-old woman with a week of left lower abdominal pain, high fever, tenderness, rebound, elevated white blood cell count, and CRP.
30	Easy	Acute appendicitis	42-year-old with right lower pain, guarding, and high CRP.
31	Moderate	Retroperitoneal hematoma	A 37-year-old male with right lumbar pain, ecchymosis, tachycardia, hypotension, and low hemoglobin after a traffic accident.
32	Easy	Splenic laceration	A 19-year-old male with left upper abdominal pain, tenderness, redness, hypotension, tachycardia, and low hemoglobin after a fall from height.
33	Moderate	Perianal abscess	A 23-year-old male with severe anal pain, difficulty sitting, fever, perianal redness, tenderness, fluctuation, leukocytosis, and elevated CRP.
34	Easy	Aortic dissection	An 81-year-old male presents with sudden severe chest pain radiating to the back, hypertension, pulse differences, and elevated D-dimer levels.
35	Moderate	Postoperative abscess	A 25-year-old male with severe pain, fever, chills, tenderness, swelling, and erythema at the surgical site after gallbladder surgery, with leukocytosis, elevated CRP, and low albumin.
36	Moderate	Renal laceration	A 30-year-old female with right-side pain after a motorcycle accident, abdominal swelling, tenderness over the right kidney, high urinary erythrocytes, and low hemoglobin.
37	Difficult	SMA aneurysm rupture	A 75-year-old male with sudden epigastric pain, rebound, hypotension, tachycardia, low hemoglobin, and metabolic acidosis.
38	Difficult	Abdominal abscess	A 65-year-old male with left lower quadrant pain, tenderness, recent abdominal surgery, elevated CRP, and leukocytes.
39	Easy	Acute cholecystitis	40-year-old with right upper quadrant pain, fever, Murphy’s sign, and elevated CRP.
40	Easy	Liver subcapsular hematoma	A 22-year-old female with right upper abdominal pain, tenderness, redness, swelling, hypotension, tachycardia, low hemoglobin, and mildly elevated liver enzymes after a fall from height.
41	Difficult	Perforated cholecystitis	A 77-year-old female with right upper quadrant pain, positive Murphy’s sign, and elevated ALP, GGT, bilirubin, leukocytes, and CRP.
42	Easy	Nephrolithiasis	A 52-year-old male with severe left-sided pain, red urine, and kidney tenderness.
43	Moderate	Peritonitis	A 58-year-old female with abdominal pain, swelling, fever, recent paracentesis, tachycardia, and elevated CRP and leukocytes.
44	Easy	Choledocholithiasis	A 70-year-old female with severe right upper quadrant pain, jaundice, tenderness, leukocytosis, and elevated bilirubin and alkaline phosphatase levels.
45	Difficult	Emphysematous cholecystitis	An 86-year-old woman with 48 h worsening right upper quadrant pain, fever, nausea, and vomiting. Exam shows Murphy’s sign; labs reveal leukocytosis, elevated CRP, and bilirubin.

SMA, superior mesenteric artery; SMV, superior mesenteric vein; CRP, C-Reactive Protein.

## Data Availability

The datasets generated and/or analyzed during the current study are not publicly available due to Local Ethics Committee policies but are available from the corresponding author upon reasonable request.
